# Dr. Cuming, on Refrigeration in Fever

**Published:** 1807-09

**Authors:** Ralph Cuming


VOL. XVIII.] SEPTEMBER, 1807. [NO. 103.

*Printed for R. PHILLIPS, by W. Thorne, Red Lion Court, Fleet Street, London*

### *To the Editors of the Medical and Physical Journal*

GENTLEMEN,

AS it is a matter of the very highest importance, that medical improvements should be made as public as possible, as well for the credit and benefit of the profession, as the good of the community; I am happy in embracing the earliest opportunity of transmitting, for the information of your readers, the following Extract of a Letter, addressed by me to the Transport Board.

"HONOURABLE SIRS,

"In my last, I did myself the honour of communicating to you a new method which I had adopted, of refrigerating patients, ill of the ardent fever of Tropical Climes; I have now the pleasure to acquaint you, that I have improved upon the plan, and can in the space of a few minutes, so effectually cool a sick person, that he shall complain of an icy coldness, instead of a burning heat.—Therefore, the grand desideratum of reducing the temperature of the body to its natural and healthy standard being so easily effected, I have every reason to think, from the great experience I have had, that the fevers of this country will be brought more under the controul of medicine than they have ever *yet been;* for every medical Tyro knows that violent vascular action, and an increased degree of caloric, or matter of heat, soon destroy the principium vitæ, or what has been termed excitability, and kill the patient.

"The mode of refrigeration is as follows: I order the sick to be either sprinkled or sponged with ardent spirit; and though common rum of the country is what I have used, the higher the proof the more expeditiously will its effects be produced. I hope the simplicity of the thing will not, in the estimation of the Board, detract from its merits, for merit it certainly has, and will be the means, under the

Divine Blessing, of saving the lives of thousands of those brave men, on whom England places such great dependance. Had I time, and were I placed in a situation to do it, I would transmit the minutiæ of my practice; but what I have already pointed out, is the grand and leading principle of it, the basis on which every other indication rests. I have been very successful in allaying sickness and vomiting; blistering the epigastrium, opium, aq. menthæ pip. with the saline mixture in the act of effervescence, are remedies I use; the alkaline solution sweetened, I have put up in one bottle, and fresh lime juice in another; they are mixed almost in contact with the *lips* of the patient, and given instantly, before the carbonic acid gas has time to escape. It is not to be expected that every patient can be saved by any method, however improved it may be; yet, if one in ten cases can be preserved who otherwise would have died, the matter becomes a national object, and demands the serious and attentive consideration of the chief officers of the medical department, and of others whose talents and situations enable them to judge and appreciate, as well as to represent and make public what they conceive will benefit the service. I found the effect of the cold affusion to be so very transitory, that there was a necessity of continuing it every ten minutes, and after all, the fever would run on for days, continually wearing out the patient, losing time and himself. The refrigerating plan, with wet sheets, mentioned in my last, had a more expeditious and salutary effect. The method I have now had the honour of submitting to your consideration, I conceive to have arrived at the very acme of perfection. One pint of spirit is sufficient for one refrigeration; and three, will generally lower the pulse, and abstract redundant heat."

*H. M. Naval Hospital, Antigua, June* 7, 1807.

"I have great pleasure in stating to the Board, that the happiest effects continue to result from the application of rum; all the surgeons who have been made acquainted with this mode of refrigeration, are delighted with it; and such as have had an opportunity of trying it, declare that they have cured men in a few hours, who might otherwise have died, or remained ill for a long time; there is no doubt of the cold affusion being entirely superseded by its use, nor of the practice being generally adopted here, and elsewhere.

"As every thing is to be done on the first attack, and the surgeons are armed with so powerful an antidote, it is to be hoped hoped that the mortality hitherto so prevalent, will at length be very considerably arrested in its progress.

"Before concluding, I beg leave to mention a remedy which I have discovered against Ulcus Gangrenosum, which might hold a place in a Dispensatory, under the name of Pulvis Antiphagedænicus, vel Pulvis Cinchona Nitratus Opiatus.

"℞. Pulv. Cinchona ℥i. Pulv. Sal. Nitri. Pulv. Opii aa ʒi. M. ft. Pulvis inspergendus super ulcere quoties opus fuerit. The decoctum cinchona with some of the compound tincture is to be taken at the same time, and continued until the ulcer be healed.

"I am, &c.

RALPH CUMING, M. D."

---

